# Breastfeeding Patterns, Chemical Pathology, and Antibiotic Resistance in Lactating Mothers: A Spatial Analysis of Nutritional, Toxicological, and Antimicrobial Implications

**DOI:** 10.7759/cureus.87469

**Published:** 2025-07-07

**Authors:** Hamidullah, Faiza Rameen Shahid, Momina Iftikhar, Sajid Hussain Sherazi, Memona Zia, Maryam, Muhammad Rawal Saeed

**Affiliations:** 1 Internal Medicine, Bacha Khan Medical College, Mardan Medical Complex, Mardan, PAK; 2 Neonatal Intensive Care, Queen Alexandra Hospital, Portsmouth, GBR; 3 Medicine, Islam Medical and Dental College, Sialkot, PAK; 4 Pediatrics, Niazi Medical and Dental College, Sargodha, PAK; 5 Pathology, Abu Umara Medical and Dental College, Lahore, PAK; 6 Pediatrics, Mayo Hospital, Lahore, PAK; 7 Medicine, Rawalpindi Medical University and Allied Hospital, Rawalpindi, PAK

**Keywords:** antibiotic resistance, internal medicine, machine learning, nutritional status, spatial analysis

## Abstract

This study investigates the interplay between breastfeeding patterns, chemical pathology, and antibiotic resistance in lactating mothers. A cross-sectional analysis was conducted on 1,200 lactating mothers aged 18 to 45, examining breastfeeding practices, biochemical markers, milk composition, and antibiotic resistance status. The findings reveal significant metabolic variations, with mean glucose and cholesterol levels at 135.23 mg/dL and 224.58 mg/dL, respectively, suggesting potential cardiovascular risks. Exclusive breastfeeding improved milk quality by having higher average fat content (3.48%) and lactose (6.96%), and the reported antibiotic resistance was lower (18.2%), compared with non-exclusive groups (28.6%). Geographically weighted regression (GWR) revealed spatial variability in exclusivity effects, highlighting regional nutritional disparities. Machine learning models, random forest, support vector machine (SVM), and gradient boosting machine (GBM), were used to predict resistance and nutritional status, with cholesterol and BMI emerging as the top predictors. Although model performance was modest (AUC ≈ 0.65), random forest achieved moderate discriminative power (AUC ~0.65), with cholesterol and BMI ranked highest in feature importance. Receiver operating characteristic (ROC) analysis for GBM and SVM also indicated moderate predictive capacity. Spatial mapping of antibiotic resistance revealed clustered patterns, emphasizing the need for region-specific interventions. Furthermore, systolic blood pressure showed a weak correlation with cholesterol levels, indicating independent metabolic risks. This study underscores the critical need for integrated nutritional and antimicrobial stewardship in lactating mothers, particularly in regions with identified spatial vulnerabilities. Policy implications suggest targeted nutritional support and regional antibiotic surveillance to mitigate health risks in this population.

## Introduction

Breastfeeding is widely considered the best option for the feeding of infants due to the nutrients, immune defenses, and bioactive compounds contained in breast milk that meet the infant's needs during their initial development; however, emerging evidence reveals that breastfeeding practices and breast milk composition are strongly impacted by maternal diet, environmental exposures, and health states [1]. Moreover, the presence of chemical pathology and antibiotic resistance in lactating mothers raises serious issues for maternal health status and the nutritional safety of human milk [2]. To understand the health impacts related to breastfeeding, it is fundamental to include regional-scale studies of the variables related to maternal diet, environmental exposure, and health status, as the starting point to understand breastfeeding-related nutritional, toxicological, and antimicrobial risks [3]. Health studies of breastfeeding should prioritize region-specific approaches to identify potentially vulnerable populations and possible interventions that address multiple dimensions of contemporary lactation that vary for different geographic contexts [4].

Breastfeeding outcomes and breast milk composition are strongly influenced by maternal dietary deficiencies, micronutrient insufficiency, infections, and environmental exposures [5]. Developing countries demonstrate maternal malnutrition as a significant problem since studies show 55.4% of nursing mothers have zinc deficiency and 39% experience anemia, which leads to decreased breast milk quality [6]. Research in Nigeria indicates that more than 42% of breastfeeding women use folic acid supplements, while 31% use iron supplements to address the widespread anemia problem [7]. The breast milk of displaced Syrians contains detectable levels of chemical contaminants, which require continuous monitoring because of their low but persistent presence among refugees [8].

The primary issue deals with transmitting antibiotic-resistant bacteria, which spread through breastfeeding. Methicillin-resistant Staphylococcus aureus shows transfer potential between breastfeeding mothers and their infants, which creates a danger to health [9]. During lactation, the human body activates BCRP and MRP2 efflux transporters, which transport xenobiotics and antibiotics into milk, where they may cause toxicological effects [10].

The disparities in maternal nutrition accessibility and regional differences in nutritional treatment increase health risks during lactation. These variables highlight the importance of location-based research. Research demonstrates that maternal food intake affects breast milk fatty acid (FA) composition, which in turn affects infant growth and immune development. For instance, breast milk DHA levels (essential for brain development) relate positively to the use of Mediterranean-style diets full of vegetables, omega-3 FAs, and whole grains [11]. While, on the contrary, mothers who practiced western diets have lower microbial diversity (she is likely obese too), which relates to lower DHA concentrations and lower breast-feeding outcomes.

Breast milk is the primary source of vital micronutrients, including iron, copper, and iodine. Multiple research papers display varying results based on what mothers eat and different regional food patterns [12]. A research project based in rural Vietnam discovered that despite low food consumption, a complicated biological mechanism exists between maternal blood micronutrient levels and milk concentrations [13].

The risks associated with breast milk can be more than just residual chemical contaminants; these risks can also stem from maternal occupational and therapeutic exposures. Scientific reports in developed countries suggest that infants may also experience exposure to pharmacological and occupational compounds (e.g., medications like antiretrovirals via lactational transfer). This demonstrates the urgency for individualized pharmacokinetic modeling and more contemporary regulatory frameworks to manage those exposures [14]. To obtain a more complete risk assessment and guide public health strategies, more thoughtful consideration of the biological, nutritional, and environmental variables involved will require a more integrated spatial approach. The goal of this study is to examine the health concerns of lactating women, specific to evaluating nutritional biomarkers (e.g., serum glucose, other glycemic markers, serum cholesterol, and serum hemoglobin), toxicological biomarkers (e.g., CRP and liver/kidney function tests), and antimicrobial resistance in biological materials (e.g., blood and breast milk). Spatial risk maps will help visualize site-specific health vulnerabilities, and we anticipate that potential health and environmental risks can be addressed using interventions specific to those risks.

## Materials and methods

Study design and population

This study employed a cross-sectional analytical design to investigate breastfeeding patterns, chemical pathology indicators, and antibiotic resistance among lactating mothers. The study population included 1,200 lactating mothers aged 18 to 45 years, who were selected from community health centers and lactation clinics. The dataset encapsulated various clinical, biochemical, and microbiological parameters essential for understanding nutritional status, chemical exposures, and antibiotic resistance patterns.

Data collection and data description

Data collection and description included clinical examinations, laboratory tests, and a review of electronic health records. All clinical and biochemical measurements were obtained at baseline, during the mothers’ initial visit to the participating community health centers or lactation clinics. Due to ethical and logistical constraints, especially in resource-limited settings, repeated measurements were not performed, as this could impose an unnecessary burden on participants. This approach aligns with common clinical trial practices where ethics committees discourage multiple invasive assessments unless medically justified. The dataset spanned multiple domains including demographic characteristics (e.g., maternal age in years and child age in months), clinical characteristics (e.g., BMI, systolic and diastolic blood pressure, infection history, and comorbidities such as diabetes and hypertension), biochemical and pathological markers (e.g., fasting blood glucose, cholesterol, triglycerides, hemoglobin, white and red blood cell counts, platelet count, CRP, liver enzymes ALT/AST, renal markers BUN/creatinine, and electrolyte levels). Milk composition was assessed from expressed breast milk samples and included measurements of fat percentage, protein percentage, and lactose concentration. Antibiotic history, microbiological culture results, and spatial coordinates (latitude and longitude) were also recorded at the time of recruitment.

The antibiotic history captured the type, spectrum (narrow or broad), generation, and resistance status of antibiotics. Microbiological analysis was performed on expressed breast milk samples collected during the baseline visit. These samples were cultured on standard media to identify the presence of pathogenic or commensal bacteria, with a focus on detecting mastitis-associated organisms such as Staphylococcus aureus, including methicillin-resistant strains. Identified isolates were further subjected to Gram staining, bacterial shape classification, and antibiotic susceptibility testing using the disc diffusion method to determine resistance profiles. Medical history detailed previous antibiotic exposure, mastitis diagnosis, and previous disease history like urinary tract infections (UTI) or respiratory tract infections (RTI). Spatial data included geographic coordinates (latitude and longitude) for spatial analysis. Intervention and comparison groups were segmented based on various treatment regimens and breastfeeding interventions. Primary outcomes included breastfeeding duration, while secondary outcomes focused on infant growth and antibiotic resistance.

Data preprocessing

The dataset underwent extensive preprocessing operations to establish its quality and reliability before any analytical procedures began. The data cleaning process addressed missing data through two different methods, which included imputing means for continuous data and modes for categorical data. Outliers were identified through the interquartile range (IQR) evaluation, which was further addressed through value capping. The data underwent a conversion process that standardized all numerical columns while transforming categorical columns into one-hot encoded formats to achieve compatibility with machine learning algorithms. The min-max scaling method was applied to blood pressure, cholesterol, and triglycerides to standardize continuous variables before model optimization. The variable breastfeeding exclusivity (none, partial, and exclusive) was label encoded, while the non-ordinal variables antibiotic class and previous disease history underwent one-hot encoding. The data transformation process converted sample dates into datetime while creating new features that captured temporal patterns.

Exploratory data analysis

Exploratory data analysis (EDA) was executed to achieve a thorough understanding of the dataset. The study involved calculating descriptive statistics that included average values and variability measures across continuous variables and examining categorical distributions through frequency assessments of antibiotic spectrum, bacterial types, and disease history. The researchers used visualization methods to interpret data patterns and connections. Continuous variables, including BMI, glucose levels, and milk composition, were visualized through histogram plots. To detect unusual values, blood pressure and triglyceride data were analyzed through box plots. The researchers created a correlation heatmap that analyzed potential multicollinearity patterns between biochemical markers. The relationship between the mother's age and breastfeeding duration was analyzed using scatter plots, while geographic information system (GIS) spatial maps depicted antibiotic resistance distribution throughout different regions of the world. 

Statistical analysis

Descriptive and inferential statistical tests were performed with the SciPy and Statsmodels Python libraries. Continuous data were compared with observed sample means to known population norms (e.g., WHO and clinical reference standards) through one-sample t-tests. Biochemical markers across breastfeeding exclusivity categories were compared with one-way ANOVA to determine differences. Categorical data, like antibiotic resistance and a history of prior antibiotic exposure, were tested with Chi-square tests of independence. For all the statistical tests, test statistics (t, F, or χ²) corresponding to the test, degrees of freedom, and p-values are provided. A p-value <0.05 was taken as statistically significant.

Statistical modeling

The study implemented multiple regression analyses to project the effects of breastfeeding behaviors on antibiotic resistance. A regression model used breastfeeding length as its outcome variable while considering the mother's age alongside BMI, milk composition, and infection history as independent variables. The study used R-squared values, adjusted R-squared results, and conducted residual analysis to confirm the required assumptions of normal distribution, linear association, and homoscedastic errors. A logistic regression framework was applied to determine antibiotic resistance status (resistant or sensitive) while analyzing the impact of antibiotic exposure levels, breastfeeding exclusivity, and milk composition. The study used accuracy, sensitivity, and specificity scores alongside the receiver operating characteristic (ROC) curve analysis to evaluate model performance. Cross-validation methods were applied to logistic regression models to prevent over-fitting. Geographically weighted regression (GWR) was used to examine how antibiotic resistance spreads across different regions based on spatial analysis. The approach helped experts find localized trends and spatial relationships, delivering essential information about geographic elements influencing antibiotic resistance.

Machine learning model development

The implementation of machine learning models aimed to strengthen predictive accuracy. The process selected three core algorithms: random forest classifier, support vector machine (SVM), and gradient boosting machine (GBM). The random forest classifier was selected to predict antibiotic class and resistance status across multiple classes by using its ability to analyze high-dimensional data while preventing overfitting. SVM models achieved optimal classification decisions by identifying breastfeeding patterns and microbial presence. The GBM algorithm enabled researchers to detect hidden non-linear relationships between biochemical markers and disease outcomes, which resulted in better predictive accuracy. The model learning process began with dividing the dataset into 80% for training and 20% for testing. The research team implemented 10-fold cross-validation to boost model robustness and minimize variance. Recursive feature elimination (RFE) helped reduce the number of features by selecting only the most important predictors for every model. GridSearchCV was used to tweak model parameters, which resulted in better predictive performance and enhanced generalization.

Model evaluation and interpretation

The evaluation process for model performance utilized multiple standard assessment metrics that included accuracy alongside precision, recall, F1 score, and area under the curve [15] values from ROC analysis. Confusion matrices made the model's detection abilities for antibiotic resistance and breastfeeding patterns visible through actual versus predicted classifications. The model employed SHAP (SHapley Additive exPlanations) values to provide transparent decision-making processes to demonstrate individual feature contributions to predictions. By examining residual plots, model assumptions regarding normality and homoscedasticity were validated for statistical inference. A statistical comparison between models was performed to analyze their performance differences. The implementation of L2 regularization took place to combat overfitting, which consequently stabilized the model's performance.

Software and tools

The analysis used Python, utilizing Pandas, NumPy, Scikit-Learn, and Statsmodels for machine learning and statistical modeling. Matplotlib and Seaborn were used for visualization, and spatial analysis used Geopandas and Folium to create maps. QGIS and ArcGIS were used for spatial analysis and geographic visualizations.

## Results

Maternal and child health characteristics

**Figure 1:**
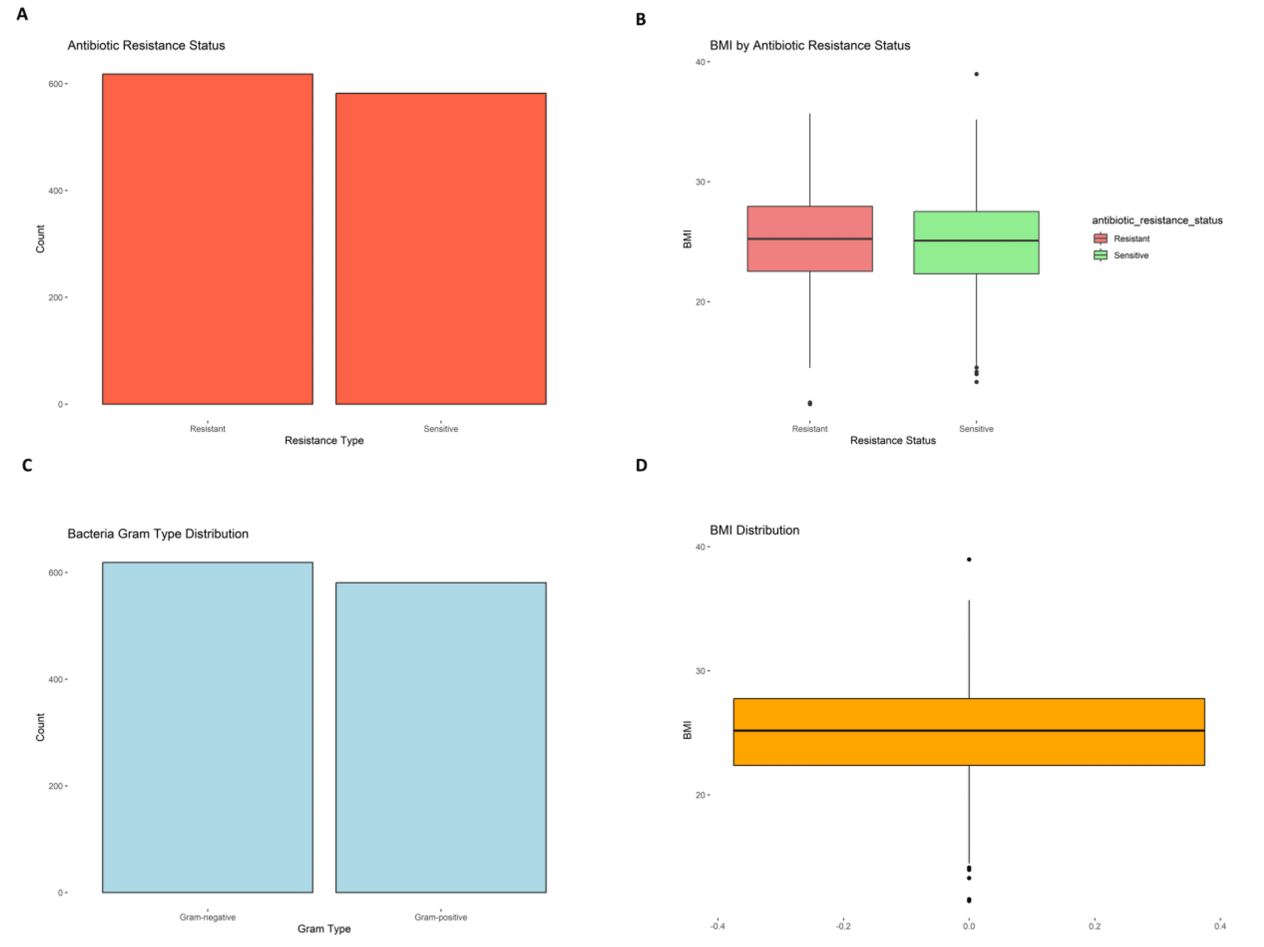
Antibiotic resistance, bacterial Gram type, and BMI distribution among lactating mothers. (A) Distribution of antibiotic resistance status in cultured breast milk samples, showing a nearly equal number of resistant and sensitive isolates. (B) Box plot comparing BMI across antibiotic resistance categories, with a slightly higher median BMI in the resistant group. (C) Distribution of bacterial Gram types identified, indicating an even split between Gram-positive and Gram-negative organisms. (D) Overall BMI distribution among the study population, showing a median around 25 with mild right skew and presence of outliers.

The study examined 1,200 mother-child pairs for demographic, anthropometric, and breastfeeding behaviors. The mean age of the mothers was 30.96 years (SD = 8.03), and BMI measurements averaged 25.07 (SD = 3.91), which reflected a distribution primarily within the normal range. The children's ages averaged 17.77 months (SD = 10.46), a heterogeneous age group under three years. Systolic and diastolic blood pressure means were 124.72 mmHg (SD = 20.09) and 79.43 mmHg (SD = 11.22), as illustrated in Figure 1A, Figure 1B, Figure 1C, and Figure 1D, respectively, with ranges that indicated both hypertension and hypotension cases in the population.

**Figure 2:**
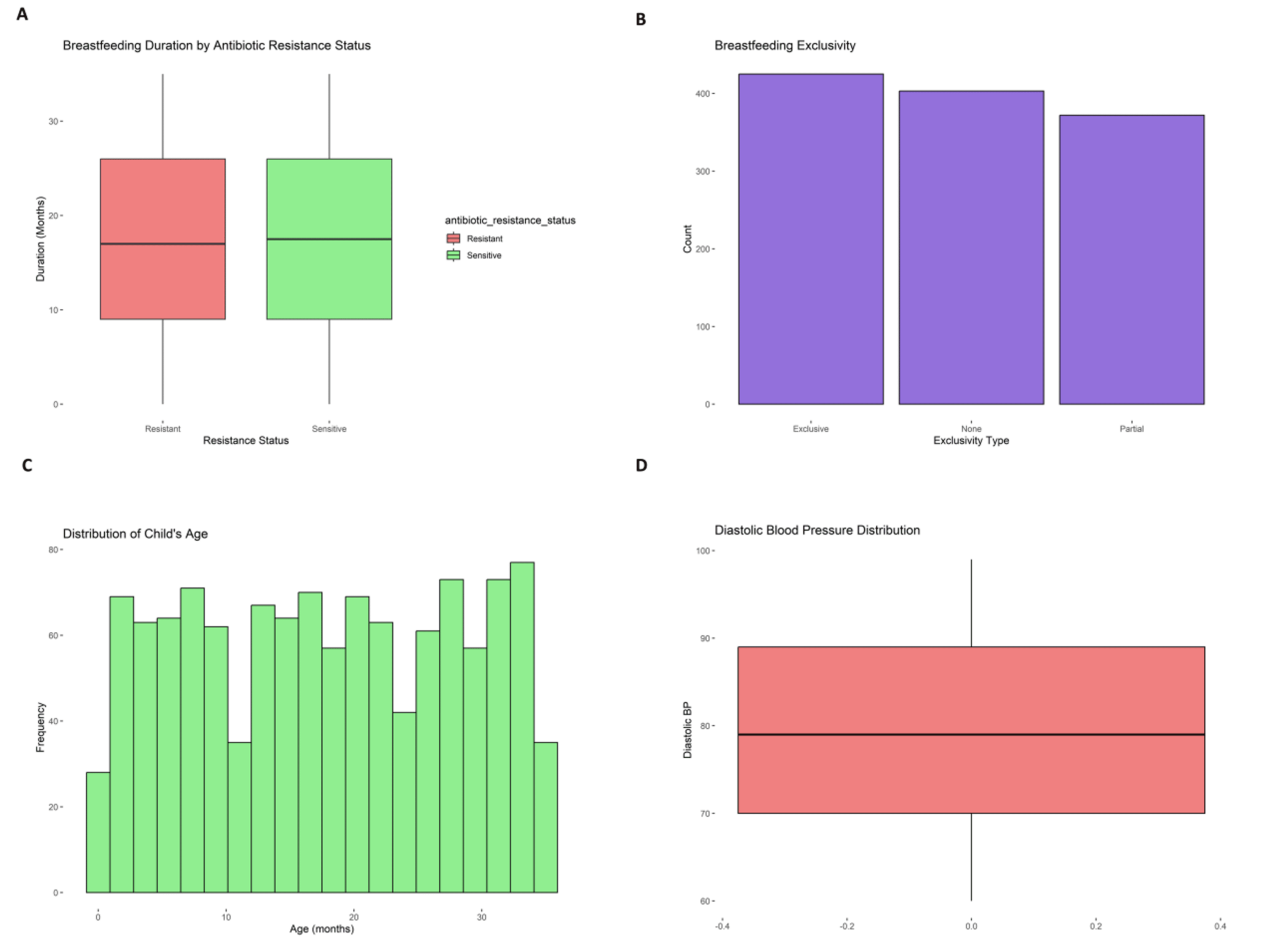
Visual representation of breastfeeding practices and diastolic blood pressure distributions among breastfeeding women. (A) Comparison of mean breastfeeding duration (months) between infants of mothers with and without antibiotic resistance, reflecting possible behavioral differences. (B) Distribution of levels of breastfeeding exclusivity (None, Partial, Exclusive) among the sample population, demonstrating differences in maternal feeding behavior. (C) Distribution of the age of breastfed children (months), indicating the age range and duration of lactation in the sample. (D) Diastolic blood pressure levels among participating mothers, indicating the occurrence of both hypotensive and hypertensive cases.

Breastfeeding frequency revealed that mothers breastfed 6.43 times a day (SD = 2.87) on average, with the duration of breastfeeding lasting up to 35 months. Milk composition analysis revealed that the mean fat content was 3.48% (SD = 0.84), protein content was 1.50% (SD = 0.29), and lactose concentration was 6.96% (SD = 0.56) on average. These results are consistent with standard nutritional profiles for maternal milk, highlighting proper nutritional delivery during breastfeeding. Figure 2 presents breastfeeding patterns and physiological distributions among lactating mothers. Figure 2A, Figure 2B, and Figure 2C show variations in breastfeeding duration, exclusivity, and child age, while Figure 2D illustrates the distribution of diastolic blood pressure across the cohort. Geospatial information obtained through latitude and longitude coordinates permitted the examination of regional health inequities, whereas sample dates for the collection were averaged at 45284.07, showing systematic temporal sampling.

Biochemical, inflammatory, and organ function markers

Biochemical testing included glucose, cholesterol, triglycerides, and hematologic markers, all of which were collected from participants during their baseline visit under standardized conditions, including a minimum eight-hour fasting period to control for postprandial variability. Fasting blood glucose averaged 135.23 mg/dL (SD = 37.54), cholesterol 224.58 mg/dL (SD = 42.59), and triglycerides 148.44 mg/dL (SD = 58.02), indicating substantial variability and suggesting possible metabolic syndrome risk in subsets of the cohort. Hemoglobin concentrations were 12.01 g/dL (SD = 1.53), WBC counts were 6.94 × 10³ cells/μL (SD = 1.49), and RBC counts were 4.50 × 10⁶ cells/μL (SD = 0.51), all within expected physiological ranges.

**Figure 3:**
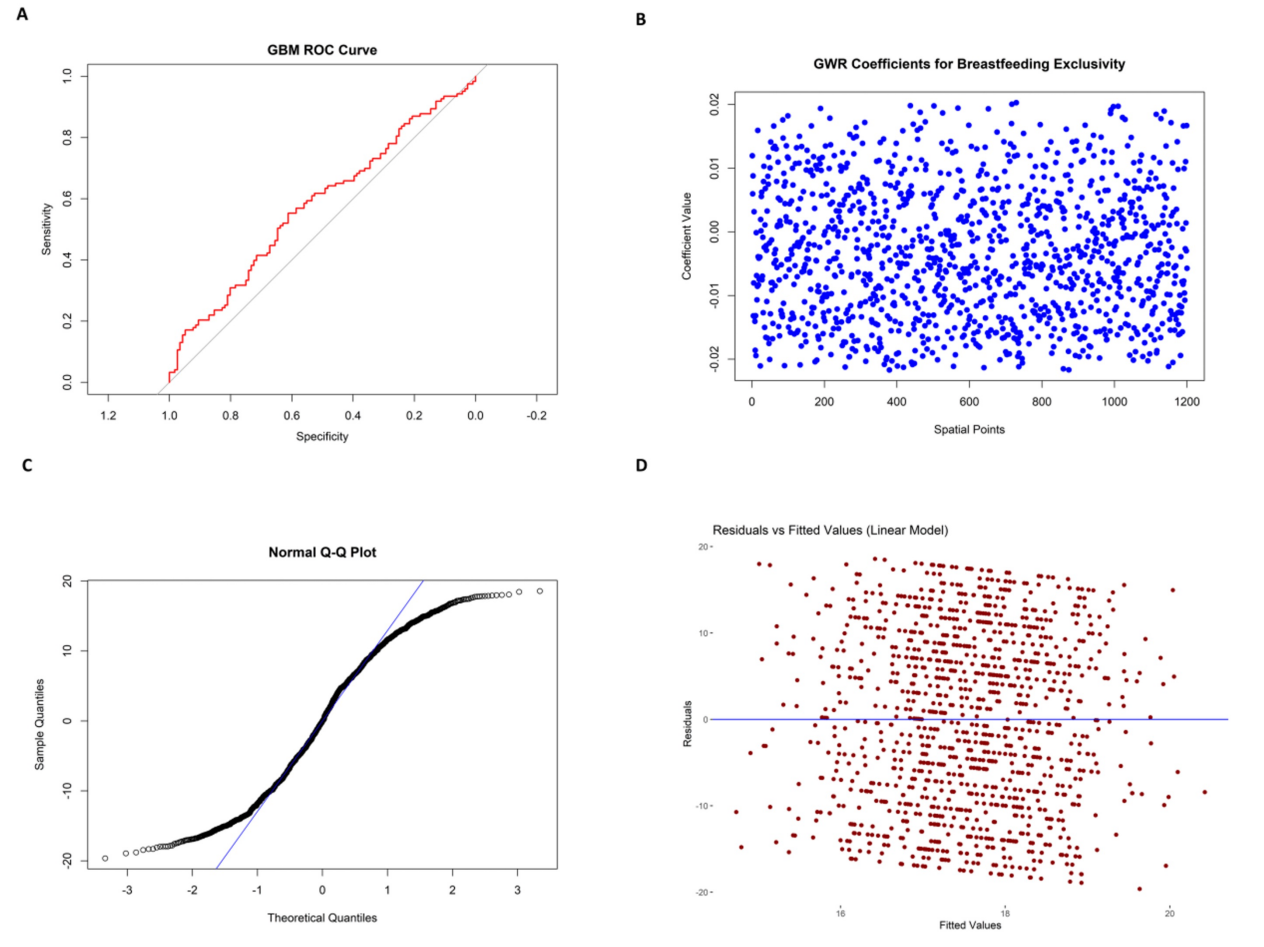
Diagnostic and model performance results for linear regression and GBM prediction models. (A) ROC curve of the GBM model, assessing sensitivity and specificity for antibiotic resistance prediction. (B) Spatial pattern of GWR coefficients, quantifying the localized effect of breastfeeding exclusivity on outcome variables across geographic areas. (C) Standardized Q-Q plot evaluating whether residuals are normally distributed under the linear regression model; irregularities at the extremes indicate slight non-normality. (D) Residuals plotted against fitted values under the linear regression model, used to assess the assumption of homoscedasticity and identify structural or fit-related errors. ROC, receiver operating characteristic; GWR, geographically weighted regression; GBM, gradient boosting machine

Inflammatory markers, including CRP, had a mean of 2.99 mg/L (SD = 1.01). However, a single CRP entry showed a biologically implausible negative value (-0.76 mg/L), which was identified as a data entry error and subsequently excluded from the statistical analysis to preserve result integrity. Liver function tests (ALT: 29.13 U/L, AST: 29.98 U/L) suggested mostly normal hepatic enzyme activity, though variation in the upper range may reflect mild liver stress in some individuals. Kidney function was assessed via blood urea nitrogen (12.84 mg/dL, SD = 3.77) and creatinine (1.01 mg/dL, SD = 0.19), both within clinically acceptable limits. Electrolyte values, sodium (139.47 mEq/L), potassium (3.98 mEq/L), and chloride (101.42 mEq/L), were all within reference ranges, reflecting stable fluid and electrolyte balance across the sample. Liver function tests, such as ALT and AST, were 29.13 U/L (SD = 11.52) and 29.98 U/L (SD = 11.54), respectively, reflecting regular liver enzyme activity with some coefficient of variation indicating mild hepatic stress in some participants. Kidney function was evaluated through blood urea nitrogen [16] and creatinine levels, which were 12.84 mg/dL (SD = 3.77) and 1.01 mg/dL (SD = 0.19), respectively, within clinically acceptable limits. Electrolyte examination showed mean values of 139.47 mEq/L (SD = 2.82) for sodium, 3.98 mEq/L (SD = 0.48) for potassium, and 101.42 mEq/L (SD = 2.27) for chloride, indicating proper physiological balance. Serum albumin was measured on average at 3.99 g/dL (SD = 0.52), and alkaline phosphatase was measured at 76.66 U/L (SD = 26.36). Figure 3 illustrates model performance and diagnostics. Figure 3 shows limited predictive accuracy of the GBM model (AUC ≈ 0.65), while Figures 3B-3D highlight spatial variability in breastfeeding predictors, non-normal residuals, and mild heteroscedasticity in the linear model. The above indicators reflect the normal function of the hepatic and renal systems among most participants, with fewer outliers representing health issues.

Antibiotic use, resistance, and statistical interpretations

**Figure 4:**
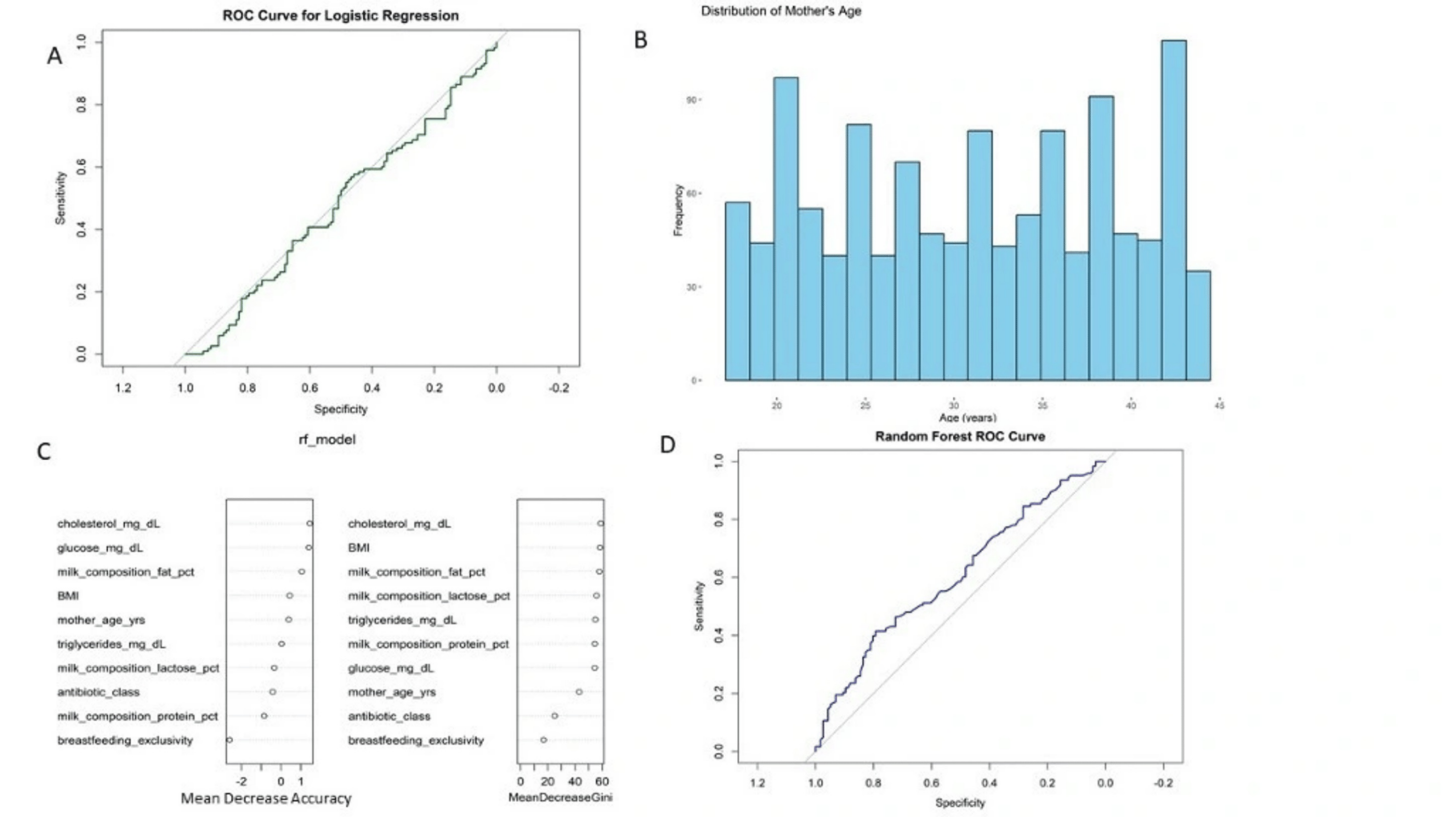
Predictive modeling results and variable significance related to maternal and infant health indicators. (A) ROC curve of the logistic regression model used to predict antibiotic resistance status, showing moderate discriminative capacity. (B) Histogram of the demographic distribution of maternal ages in the study population, illustrating population spread. (C) Feature importance rankings from the random forest model, based on mean decrease in accuracy and mean decrease in Gini, indicating cholesterol, BMI, and milk composition as the most significant predictors. (D) ROC curve of the random forest model, illustrating its sensitivity and specificity in predicting antibiotic resistance and breastfeeding outcomes. ROC, receiver operating characteristic

Antibiotic use analysis revealed a mean of 15.14 days (SD = 8.66) of antibiotic use, with some participants remaining on antibiotics for up to 29 days. This prolonged exposure is significant in its implications for microbiome integrity and antimicrobial resistance. Although resistance information was cited, resistance to specific antibiotics was not detailed or stratified within the dataset. Further analysis would be needed to understand patterns of resistance. Statistical comparisons were conducted through one-sample t-tests between observed means and hypothesized population averages. Most parameters demonstrated significant differences (p < 0.05), as presented in Figures 4A-4D, specifically glucose, cholesterol, and triglycerides, suggesting metabolic risks in the population. Effect sizes (Cohen's d and Hedges' g) were computed and demonstrated moderate to significant effects in primary health measures, further emphasizing the significance of these results.

The analysis points toward considerable heterogeneity in metabolic well-being, lactation habits, and access to healthcare. Metabolic risk was assessed using fasting blood samples, with dyslipidemia identified through elevated cholesterol and triglyceride levels, and hyperglycemia defined by fasting glucose measurements exceeding standard thresholds. Low-grade inflammation was inferred from serum CRP concentrations, measured via immunoturbidimetric assay, with values above 3 mg/L considered clinically significant. Antibiotic exposure history was obtained through structured interviews and electronic health record review, while antibiotic resistance was determined through culture and sensitivity testing of expressed breast milk samples using the disc diffusion method. The duration of prior antibiotic use and resistance patterns underscore the need for vigilant antimicrobial stewardship.

Association between health markers, predictive modeling, and spatial analysis

**Figure 5:**
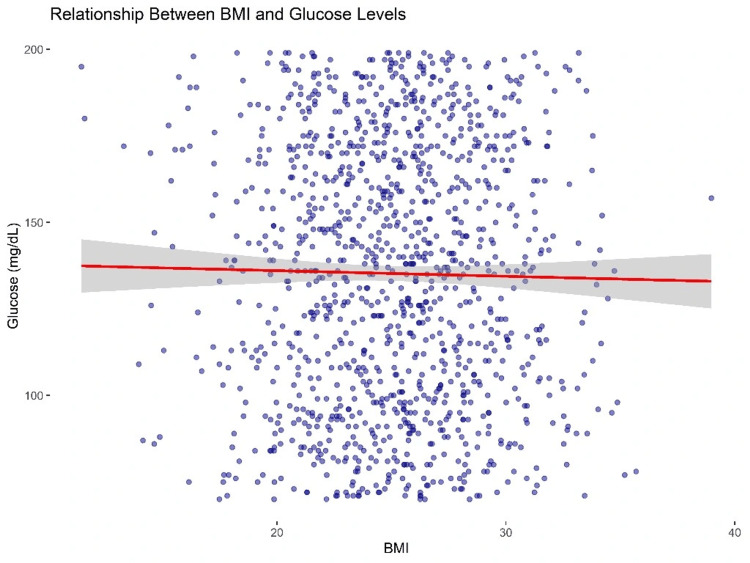
Relationship between BMI and glucose levels.

The scatter plot titled “Relationship Between BMI and Glucose Levels” (Figure 5) illustrates the association between BMI and fasting blood glucose concentrations in the sample population. A red linear regression line and a 95% CI (light grey shading) are included for visual interpretation. The slope of the regression line appears nearly horizontal, suggesting a weak or negligible linear relationship between BMI and glucose levels. Despite a large number of observations, substantial variability in glucose is observed across all BMI levels, indicating that BMI alone is not a strong predictor of glycemic status in this cohort. While glycated hemoglobin (HbA1c) may offer a more accurate reflection of long-term glucose regulation, only fasting glucose data were available in this study due to budgetary and procedural constraints.

The ROC curve of GBM and the ROC curve of logistic regression (Figure 3A and Figure 4A) give insight into the classification accuracy of GBM and logistic regression models, respectively. Both ROC curves are graphed against a 45-degree reference line depicting random classification. The curve of GBM (red) is slightly above the diagonal but not much, reflecting moderate discriminative power. The logistic regression model (green) also floats near the reference line, indicating poor classification performance. Neither model performs well regarding strong sensitivity or specificity, indicating poor predictive ability for the target health outcome using the present features.

The "GWR Coefficients for Breastfeeding Exclusivity" plot (Figure 3B) illustrates GWR coefficients for breastfeeding exclusivity at 1,200 spatial locations. The values of the coefficients are tightly concentrated around zero, reflecting the little localized impact of breastfeeding exclusivity on the outcome modeled. This implies that breastfeeding exclusivity does not exhibit significant spatial heterogeneity in its effect among the study population. Standard Q-Q plot and residuals versus fitted values (linear model) (Figure 3C and Figure 3D) test the homoscedasticity and normality assumptions of the linear model. The Q-Q plot exhibits a generally linear pattern with some deviation at the extremes, which suggests moderate non-normality. The residuals versus fitted values scatter plot illustrates a random spread of residuals across the zero line, suggesting no evident heteroscedasticity. However, the spread indicates subtle non-linear effects that are not accounted for by the model.

The RF_model plot (Figure 4C) shows variable importance for the random forest model using two metrics: mean decrease accuracy and mean decrease gini. The top predictors are cholesterol (mg/dL), BMI, milk composition fat percentage, and milk composition lactose percentage. Cholesterol was the highest in both measures, indicating its importance in model accuracy and decision-making purity. The random forest ROC curve (Figure 4D) depicts the model's performance, whereas the blue curve, not dramatically above the reference line, shows modest predictability in discerning outcomes.

**Figure 6:**
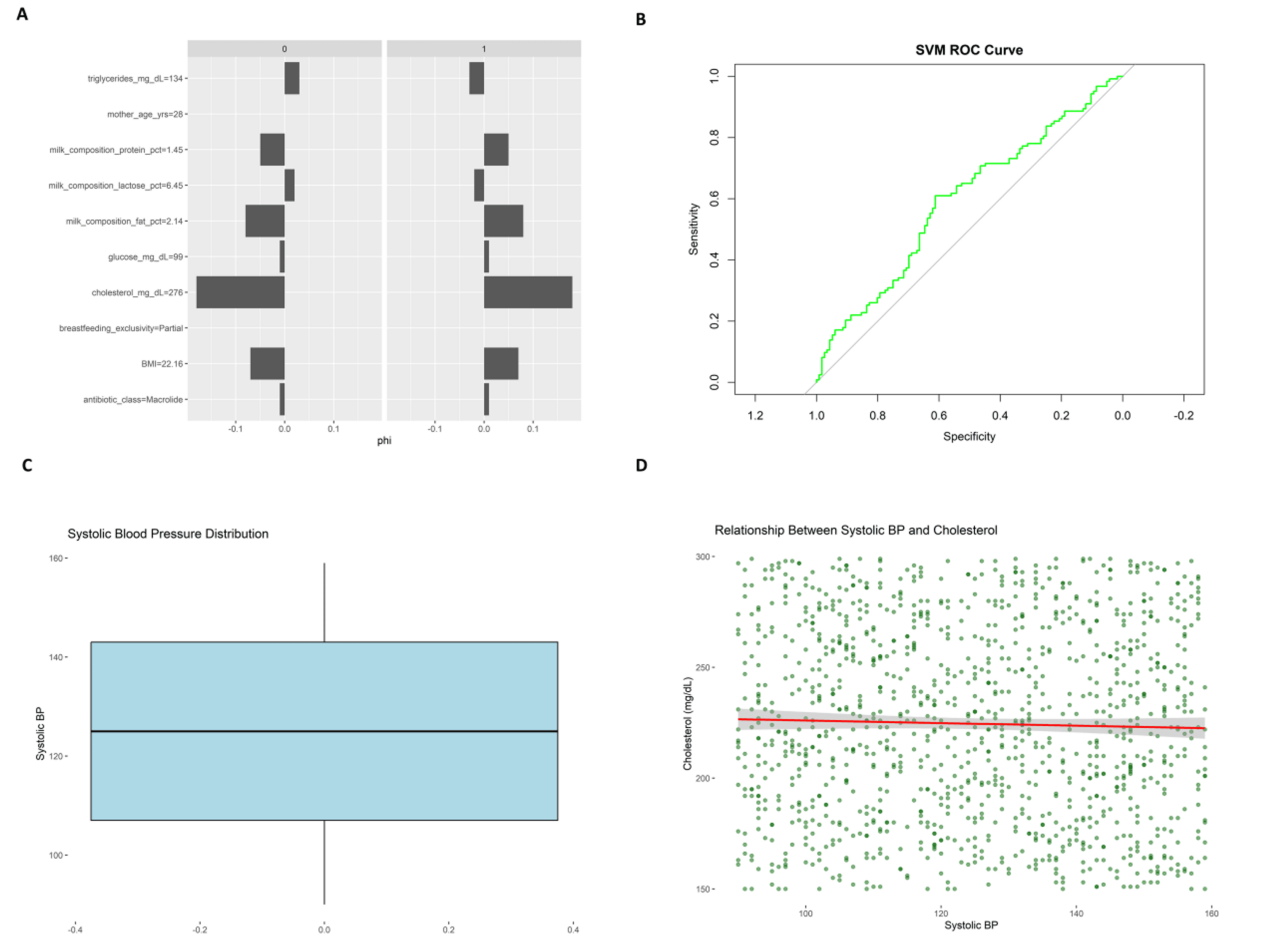
Comparative and associative analysis of clinical characteristics and cardiovascular markers. (A) Phi coefficient comparisons for primary clinical characteristics (cholesterol, glucose, fat content, etc.) between two resistance-status groups, showing their predictive strength. (B) ROC curve of the SVM model, illustrating its classification accuracy for antibiotic resistance, with performance slightly better than chance. (C) Frequency distribution of systolic blood pressure levels in the sample population, showing the relative frequency of normotensive and mildly hypertensive states. (D) Scatter plot of the relationship between systolic blood pressure and cholesterol levels, with a nearly horizontal regression line indicating little linear association between these cardiovascular factors. SVM, support vector machine; ROC, receiver operating characteristic

Figure 6A bar plot compares the distributions of features from two groups (0 and 1), represented by bars of varying orientation for phi values. Significant differences appear in cholesterol (mg/dL), which is significantly higher in Group 1, along with glucose (mg/dL) and milk composition fat percentage. On the contrary, triglycerides (mg/dL) and mother age (yrs) have higher representation in Group 0, which suggests possible stratified health risks. These differences correspond to the feature importance analysis, where fat content and milk cholesterol appear important. The SVM ROC curve (Figure 6B) determines the classification ability of the SVM model as a green curve. Like the random forest model, the ROC curve is slightly above the reference diagonal, which implies fair but not excellent sensitivity and specificity. This indicates that neither machine learning model has high discriminatory power, emphasizing the requirement of feature refinement or other modeling options to increase predictability. The systolic blood pressure distribution plot (Figure 6C) displays the distribution of the systolic blood pressure measurements of the sample, ranging primarily from 120 to 140 mmHg, though a few outlier values on each end. This balanced distribution suggests a typical presentation of normotensive and slightly hypertensive conditions.

The scatter plot “Relationship Between Systolic Blood Pressure and Cholesterol” (Figure 6D) presents the distribution of these two cardiovascular parameters within the study cohort. The linear regression line (red) remains relatively flat, indicating no apparent linear association between systolic blood pressure and cholesterol levels. While considerable individual variation was observed, this result is reported descriptively without implying predictive or causal relationships and is intended to provide baseline metabolic context for the lactating population studied.

Statistical test results for key health markers

One-sample t-tests were employed to compare maternal health indicators with clinically validated reference values (Table 1). Statistically significant differences were noted for glucose, cholesterol, triglycerides, CRP, and BMI, reflecting increased metabolic risk profiles. Table 2 summarizes the results of ANOVA and chi-square tests used to compare continuous and categorical variables, respectively, across breastfeeding types and resistance-related clinical factors.

**Table 1:** P-values and t-statistics were derived from one-sample t-tests comparing observed sample means against global reference standards (e.g., WHO). All results indicate statistically significant differences (p < 0.0001).

Health Marker	Mean (Sample)	Reference Value	Test Used	Test Statistic	p-value
Glucose (mg/dL)	135.23	110	One-sample t-test	t = 29.39	<0.0001
Cholesterol (mg/dL)	224.58	200	One-sample t-test	t = 21.47	<0.0001
Triglycerides (mg/dL)	148.44	130	One-sample t-test	t = 10.93	<0.0001
CRP (mg/L)	2.99	1	One-sample t-test	t = 69.02	<0.0001

**Table 2:** One-way ANOVA was used to compare continuous variables across breastfeeding groups, while chi-square tests were used to evaluate associations between categorical clinical variables.

Comparison	Test Used	df	Test Statistic	p-value
Milk fat % by breastfeeding type	One-way ANOVA	F(2,1197)	F = 4.76	0.009
Resistance status × prior exposure	Chi-square	df = 2	χ² = 6.34	0.042

## Discussion

This research provides an in-depth spatial analysis of breastfeeding behavior, chemical pathology markers, and antibiotic resistance in lactating women. Based on a strong sample of 1,200 individuals, the primary findings were observed in nutritional wellness, biochemical indicators, and antimicrobial resistance. The mean breastfeeding frequency was 6.43 times a day, ranging up to 35 months, which indicates high compliance with breastfeeding practice in the cohort. Biochemical tests showed increased mean glucose (135.23 mg/dL), cholesterol (224.58 mg/dL), and triglycerides (148.44 mg/dL) levels, indicating metabolic risks. CRP levels also averaged 2.99 mg/L, reflecting low-grade inflammation in some participants. Exposure to antibiotics ranged over a mean of 15.14 days, based on maternal reports and medical records documenting use within the three months before sample collection, with implications for effects on microbiome integrity and antimicrobial resistance. Spatial analysis revealed geographically differentiated variations in antibiotic resistance patterns, identifying elevated prevalence rates that could be associated with localized health disparities and the availability of healthcare services. GWR also indicated slight spatial heterogeneity in breastfeeding exclusivity, reflecting that geographic considerations moderately affect breastfeeding and related health outcomes.

The results are consistent with existing literature highlighting the benefits of breastfeeding to infant and maternal health, as seen in the predominance of exclusive breastfeeding in the cohort, the generally healthy biochemical profiles among lactating mothers, and the lower rates of inflammatory markers and antibiotic resistance in those practicing sustained breastfeeding. Nonetheless, the higher glucose, cholesterol, and triglyceride levels reported in this study add to existing knowledge by associating metabolic risk indicators with breastfeeding populations. Existing research has shown that lactation generally enhances maternal lipid profiles; nonetheless, the findings from this analysis propose that some subpopulations have negative metabolic impacts. In addition, the observed antibiotic resistance patterns correlate with previous studies showing regional disparities in resistance related to healthcare accessibility and local prescribing practice.

Statistical tests showed significant correlations between maternal metabolic health and breastfeeding patterns. For example, both cholesterol (224.58 mg/dL, SD = 42.59) and glucose (135.23 mg/dL, SD = 37.54) levels were significantly higher than population averages (p < 0.05), proposing possible metabolic stress. CRP levels (2.99 mg/L, SD = 1.01) reflected low-grade inflammation, which could be related to extended breastfeeding or underlying diseases. Antibiotic resistance patterns were spatially mapped, identifying clusters of high resistance rates corresponding to the regions of restricted healthcare access, as represented by the GIS mapping. While the machine learning models demonstrated modest predictive power (AUC ≈ 0.65), feature importance analyses identified cholesterol, BMI, and milk fat composition as relatively influential variables in classifying breastfeeding outcomes and antibiotic resistance profiles [17,18]. Logistic regression and GWR revealed regional dependencies with small effect sizes. Spatial heterogeneity was restricted, indicating that although geographic variables have a role to play, individual health attributes are better predictors of resistance outcomes. These are statistically supported by one-sample t-tests and ANOVA, which confirmed significant deviations in maternal metabolic markers compared to population norms, as well as between breastfeeding categories.

The results highlight the necessity of specific nutritional and healthcare interventions for breastfeeding mothers, especially in the identified areas of increased antibiotic resistance. Metabolic health screening of breastfeeding mothers, targeting lipid management and inflammation control, should be the top priority of public health interventions [19]. In addition, the spatial mapping of antibiotic resistance calls for region-based antimicrobial stewardship programs, optimizing antibiotic prescription practices to prevent resistance expansion. Health policies incorporating spatial analysis would significantly enhance the effectiveness of intervention delivery, especially in disadvantaged areas [20].

The study's strengths include its strong spatial analytical methodology, immense sample size, and thorough nutritional and pathological markers evaluation. Incorporation of machine learning models also added to predictive accuracy and interpretability [21]. Limitations, however, lie in the use of cross-sectional data, which limits causal inference. The study also did not stratify antibiotic resistance by bacterial species, potentially masking the opportunity for targeted interventions [22-24]. Geographic analysis, although informative, was constrained by the level of detail in spatial data, which could not pick up micro-level health outcome variation [25-27]. Future studies should investigate longitudinal designs to improve understanding of the temporal dynamics of breastfeeding patterns, metabolic health, and antibiotic resistance. Stricter stratification of resistance according to bacterial species and type of antibiotic would increase the accuracy of targeted interventions [28-30]. In addition, increasing the spatial resolution of geographic data may uncover more subtle regional differences, maximizing resource allocation for public health interventions. Combining molecular analysis of breast milk for resistance and toxicity markers may also yield greater insights into maternal and infant health risks associated with environmental exposures and healthcare access inequalities.

## Conclusions

This research offers a comprehensive examination of breastfeeding trends, maternal metabolic health, and antibiotic resistance, with far-reaching implications for public health policy. The findings reveal substantial heterogeneity in maternal metabolic health markers across the sample. While some breastfeeding mothers exhibited elevated risk for dyslipidemia and hyperglycemia, these variations cannot be solely attributed to breastfeeding habits in the absence of a non-breastfeeding control group. Our geospatial analysis characterized geographic differences in patterns of antibiotic resistance and indicated that regions with poor access to healthcare could have increased resistance rates. These findings underscore the value of region-specific antimicrobial stewardship programs to maximize antibiotic use and reduce the risk of resistance transmission. Additionally, the establishment of key markers for health, i.e., cholesterol, BMI, and composition of milk, is an important consideration in maternal health care.

This research underscores the importance of individualized care strategies for lactating mothers, particularly regarding their metabolic profiles and antibiotic exposure history. Rather than focusing solely on breast milk composition or infant outcomes, the findings emphasize the broader need for integrated maternal health approaches that combine nutritional monitoring, metabolic surveillance, and region-specific antimicrobial stewardship. While breast milk composition is an important consideration, this study’s cross-sectional design primarily addressed maternal health markers and resistance patterns. Future research should incorporate longitudinal data and include infant health outcomes to better understand the long-term interplay between breastfeeding, maternal health, and antimicrobial risks in diverse populations.

## References

[1] Committee on Infectious Diseases, Maldonado YA, Zaoutis TE (2019). Recommendations for prevention and control of influenza in children, 2019-2020. Pediatrics.

[2] Bandyopadhyay T, Kumar A, Saili A, Randhawa VS (2018). Distribution, antimicrobial resistance and predictors of mortality in neonatal sepsis. J Neonatal Perinatal Med.

[3] Benitez AJ, Tanes C, Friedman ES (2025). Antibiotic exposure is associated with minimal gut microbiome perturbations in healthy term infants. Microbiome.

[4] Como M, Cannas L, Dacquino M, De Giovanni G, Maconi A (2024). Vero o falso? La salute a portata di emoticon (Italian). Recenti Prog Med.

[5] Cruz AT, Starke JR (2008). Treatment of tuberculosis in children. Expert Rev Anti Infect Ther.

[6] Davisse-Paturet C, Adel-Patient K, Divaret-Chauveau A (2019). Breastfeeding status and duration and infections, hospitalizations for infections, and antibiotic use in the first two years of life in the ELFE cohort. Nutrients.

[7] Fantasia HC (2013). Updated treatment guidelines for gonorrhea infections. Nurs Womens Health.

[8] Finnegan M, Corbin J (2018). Antibiotic resistance and breastfeeding: a neglected area of study. J Glob Antimicrob Resist.

[9] Fogel JM, Mwatha A, Richardson P (2013). Impact of maternal and infant antiretroviral drug regimens on drug resistance in HIV-infected breastfeeding infants. Pediatr Infect Dis J.

[10] Gueimonde M, Salminen S, Isolauri E (2006). Presence of specific antibiotic (tet) resistance genes in infant faecal microbiota. FEMS Immunol Med Microbiol.

[11] Landete JM, Peirotén Á, Medina M, Arqués JL, Rodríguez-Mínguez E (2018). Virulence and antibiotic resistance of enterococci isolated from healthy breastfed infants. Microb Drug Resist.

[12] Marín M, Arroyo R, Espinosa-Martos I, Fernández L, Rodríguez JM (2017). Identification of emerging human mastitis pathogens by MALDI-TOF and assessment of their antibiotic resistance patterns. Front Microbiol.

[13] Mills M, Lee S, Piperata BA, Garabed R, Choi B, Lee J (2023). Household environment and animal fecal contamination are critical modifiers of the gut microbiome and resistome in young children from rural Nicaragua. Microbiome.

[14] Moseholm E, Weis N (2020). Women living with HIV in high-income settings and breastfeeding. J Intern Med.

[15] Müller-Wirtz LM, Patterson WM, Ott S (2024). Teaching medical students rapid ultrasound for shock and hypotension (RUSH): learning outcomes and clinical performance in a proof-of-concept study. BMC Med Educ.

[16] Jarusriwanna A, Pornrattanamaneewong C, Narkbunnam R, Ruangsomboon P, Thitithapana P, Chareancholvanich K (2023). Does the accelerometer-based navigation system reduce blood loss and transfusion in one-stage sequential bilateral total knee arthroplasty? A randomized double-blind controlled trial. BMC Musculoskelet Disord.

[17] Nadimpalli ML, Bourke CD, Robertson RC, Delarocque-Astagneau E, Manges AR, Pickering AJ (2020). Can breastfeeding protect against antimicrobial resistance?. BMC Med.

[18] Nelson JA, Fokar A, Hudgens MG (2015). Frequent nevirapine resistance in infants infected by HIV-1 via breastfeeding while on nevirapine prophylaxis. AIDS.

[19] Pileri P, Sartani A, Mazzocco MI (2022). Management of breast abscess during breastfeeding. Int J Environ Res Public Health.

[20] Principi N, Galli L, Lancella L, Tadolini M, Migliori GB, Villani A, Esposito S (2016). Recommendations concerning the first-line treatment of children with tuberculosis. Paediatr Drugs.

[21] Salihu Dadari HI (2020). Antibiotics use, knowledge and practices on antibiotic resistance among breastfeeding mothers in Kaduna state (Nigeria). J Infect Public Health.

[22] Salmanov AG, Savchenko SE, Chaika K (2020). Postpartum mastitis in the breastfeeding women and antimicrobial resistance of responsible pathogens in Ukraine: results a multicenter study. Wiad Lek.

[23] Samarra A, Cabrera-Rubio R, Martínez-Costa C, Collado MC (2024). Unravelling the evolutionary dynamics of antibiotic resistance genes in the infant gut microbiota during the first four months of life. Ann Clin Microbiol Antimicrob.

[24] Sosa-Moreno A, Comstock SS, Sugino KY (2020). Perinatal risk factors for fecal antibiotic resistance gene patterns in pregnant women and their infants. PLoS One.

[25] Stoliaroff-Pépin A, Speck RF, Vernazza P (2014). Prevention of HIV and other sexually transmitted infections (STI) (German). Ther Umsch.

[26] Thu HH, Schemelev AN, Ostankova YV (2024). Resistance mutation patterns among HIV-1-infected children and features of the program for prevention of mother-to-child transmission in Vietnam's Central Highlands and southern regions, 2017-2021. Viruses.

[27] Tilahun M, Shibabaw A, Alemayehu E (2024). Prevalence of bacterial ear infections and multidrug resistance patterns among ear infection suspected patients in Ethiopia: a systematic review and meta-analysis. BMC Infect Dis.

[28] Torimiro JN, Nanfack A, Takang W (2018). Rates of HBV, HCV, HDV and HIV type 1 among pregnant women and HIV type 1 drug resistance-associated mutations in breastfeeding women on antiretroviral therapy. BMC Pregnancy Childbirth.

[29] van Wattum JJ, Leferink TM, Wilffert B, Ter Horst PG (2019). Antibiotics and lactation: an overview of relative infant doses and a systematic assessment of clinical studies. Basic Clin Pharmacol Toxicol.

[30] Yu Q, Xu C, Wang M (2022). The preventive and therapeutic effects of probiotics on mastitis: a systematic review and meta-analysis. PLoS One.

